# Climate change adaptation strategies and their predictors amongst rural farmers in Ambassel district, Northern Ethiopia

**DOI:** 10.4102/jamba.v13i1.974

**Published:** 2021-02-17

**Authors:** Fikre Destaw, Muluken M. Fenta

**Affiliations:** 1 Department of Natural Resource Management, College of Agriculture and Natural Resources, Gambella University, Gambella, Ethiopia; 2 Department of General Forestry, Wondo Genet College of Forestry and Natural Resource, Hawassa University, Hawassa, Ethiopia

**Keywords:** adaptation, barriers, climate change, determinants, smallholder farmers

## Abstract

The present study was conducted in Ambassel district of Northern Ethiopia to understand adaptation strategies employed by rural farmers to the adverse effects of climate change and variability and factors that determine their adaptation decisions. The study was based on multistage sampling techniques to select the study villages and sampled households (HHs). Data were collected through HH survey, focus group discussions and key informant interviews. The collected data were analysed by using descriptive statistics and multinomial logit (MNL) model. The results revealed that in response to the effects of climate variability and change, the adaptation strategies deployed by farmers included terracing as soil and water conservation strategy, changing planting date, fertiliser application, crop diversification with improved variety, income diversification and livestock diversification. The result from MNL analysis showed that age, family size, educational level, farm size, income, livestock holding, access to extension, distance to market, access to climate information and agroecological zones were amongst the factors that had a significant influence on farmers’ choice of adaptation strategies. The basic barriers to climate change adaptation were lack of finance, shortage of land, inadequate climate information, lack of skill and shortage of labour. Therefore, strengthening interventions that enhance income generating activities and access to climate information should be an integral part of climate change adaptation strategies. Moreover, providing early maturing and high-value crop varieties that are more suited to the local environment is also crucial.

## Introduction

Nowadays, climate change is acknowledged as one of the most challenging and complex problem confronting the agricultural development worldwide (IPCC 2014; Tesfahunegn et al. 2016). However, agriculturural production activities in Africa are generally more vulnerable to climate change than any other socioeconomic activities (Burnett 2013; Elum et al. 2017; IPCC 2014). It is predicted that agricultural production in Africa will decrease by 8% – 22% by 2050 (Schlenker & Lobell 2010). The continuous dry seasons experienced throughout the recent 30 years and the ongoing effects of El Niño in East African nations in general and Ethiopia specifically, made food insecure to large number of people because of climate change (Yayeh 2017).

Despite the fact that the effects of climate change differ temporally and spatially, the threat to rain-fed agriculture is viewed as the most pervasive as future effects are expected to exacerbate following alteration in rainfall and temperature (Deressa et al. 2011; Kurukulasuriya and Mendelsohn 2008). Also, climate change is probably going to have an overall negative impact on the yields of main cereal crops (Deressa et al. 2008; IPCC 2014). As indicated by certain studies (Burnett 2013; Deressa et al. 2008; IPCC 2014; ISET 2013), climate change would even disrupt individuals’ daily activities, alter growing seasons, cause a decrease in crop yield and biomass production and increase risk of food insecurity. Rural farmers in low-income countries feel the adverse effects of climate change more severely.

Ethiopia is a country located in the Horn of Africa that is experiencing a warming trend of annual temperature and increasing drought severity (Burnett 2013; ISET 2013). The annual temperature of the country has been increasing by 0.37 °C every 10 years during the past 55 years (McSweeney et al. 2010). Ethiopia is one of the most vulnerable countries to climate change and variability in Africa and is frequently confronted with climate-related hazards that affect the lives and livelihoods of people (Burnett 2013; ISET 2013; World Bank 2010). Climate-related shocks and stresses with drought and flood being the major one has affected agricultural sector in Ethiopia (Deressa et al. 2011; ISET 2013). Although agriculture contributes to about 40% of gross domestic product (GDP), approximately 85% of exports and approximately 77% of total employment in Ethiopia, it is one of the most vulnerable sectors to the current and projected climate change, potentially exposing millions of people to recurrent food shortages (ATA 2017). This vulnerability is exacerbated by the existing poor socioeconomic conditions such as poor public services, population pressure, mounting poverty rate, political instability and food insecurity. Our study area, Ambassel district, northern Ethiopia is not exceptional and is adversely impacted by climate change and variability.

As Farber (2011) indicated, it is difficult to avoid climate change effects using mitigation measures. Hence, adaptation is a need as its effects manifest relatively very quickly. Also, Hassan and Nhemachena (2008) pointed that with increasing acknowledge of low adaptive capacity and vulnerability to climate effects in developing countries such as Ethiopia, the need of adaptation is so critical in the light of the fact that it can happen at macro- or microscale. Moreover, Esham and Garforth (2013) reported that the vulnerability of farmers to climate change and variability has been increasing in poor and least developing countries. This implies that adaptation measures are paramount for farmers’ well-being as agriculture is their main source of income.

Climate change has expansive ramifications to the Ethiopian farmers as most of the communities practice rain-fed agriculture. The country has started adaptation interventions against adverse effects of climate change, yet endeavours are still at a relatively early phase: it is practically more acceptable to state that the endeavours are fragmented and limited. In accordance with this, a research conducted in southern part of Ethiopia by Hurst et al. (2012) shows that adaptation interventions took place as small changes. A great part of the actual endeavours to climate change adaptations are occurring with regard to unseemly approaches amidst maladaptive practices, poor institutional frameworks and implementation practices.

Significant investigations have been carried out on climate change adaptations and their determinants in certain regions of the country (Belay et al. 2017; Deressa et al. 2011; Di Falco 2009; Tazeze et al. 2012; Tessema et al. 2013). However, none of them have focused on the South Wollo zone of Amhara regional state, Ethiopia, particularly the Ambassel district, which is the present study area. Thus, climate change adaptation strategies employed by rural farmers and their determinant factors of the study area have not been adequately assessed and documented. The lack of reported data about adaptation strategies to climate-related risks in Africa involving Ethiopia has been recognised by McSweeney et al. (2010). Besides, the greater parts of the investigations were focused on Nile Basin of Ethiopia (Deressa et al. 2011; Di Falco et al. 2009) and the studies did not address adaptation measures of rural farmers at the local level as they used regional data. To address the current research gap, the present study was conducted to identify adaptation strategies and analyse the determinants of choices of adaptation to climate change by smallholder farmers in Ambassel district, Northern Ethiopia. Evidence at microlevel is very important to introduce site-specific adaptation interventions.

## Methodology

### Description of the study area

**FIGURE 1:**
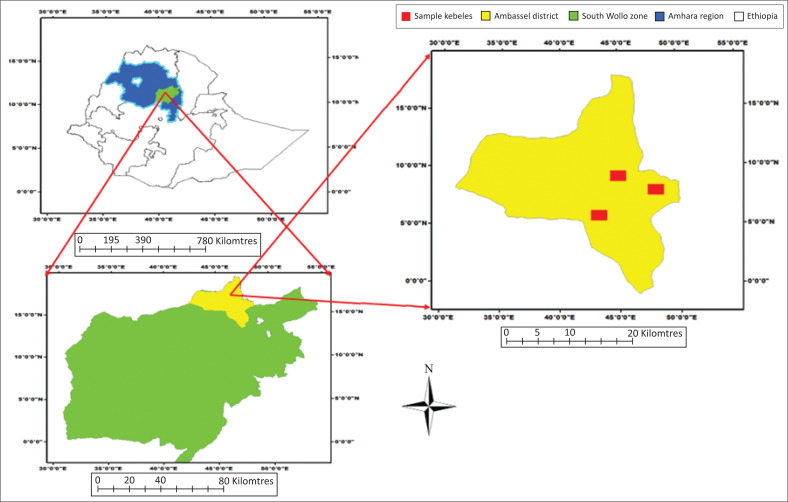
Map of the study area.

The study was carried out in Ambassel district, Amhara regional state, Ethiopia, which is located at 11º 31′ 05″North and 39º 36′ 34″East (Figure 1). Ambassel district is situated about 460 km to the North of the capital city of the country, Addis Ababa (NMA 2007). The total land area of the district is 92,699 ha (Ambassel District Office of Agriculture 2018). The altitude of the district ranges from 1425 to 3627 meters above sea level. Ambassel district receives a mean annual rainfall of 500 mm – 1500 mm in bimodal pattern. The long rainy season (*Kirmet*) occurs from June to September, and the short rainy season (*Belg*) lasts from mid-February to the end of April. The average yearly minimum and maximum temperatures are 12.5 °C and 22.5 °C (Ambassel District Office Early Warning 2018). The livelihood of the local people is mainly dependent on mixed crop-livestock production, which is mostly rain-fed agriculture.

### Sampling techniques and sample size determination

The study follows multistage sampling procedures. In the first stage, Ambassel district was selected purposively from the districts of South Wollo Zone because of the occurrence of recurrent drought in the area. In the second stage, based on the agroecological zone of the district, villages were stratified into *Dega* (Highland), *Woyina Dega* (Mid-land) and *Kola* (Lowland), and then three villages (Abet, Kollet and Walkit), one from each agroecological zone were randomly selected. The purpose of the analysis in relation to agroecological differentiation was to investigate how farmers living in different agroecological zones respond to the effects of climate variability and change. Agroecological zones are geographical regions with similar climatic conditions that reflect elevation gradients and topographic effects on temperature, rainfall and seasonality that regulate their potential to support rain-fed agriculture (Sebastian 2014). Finally, in the third stage, sample households (HHs) were selected using systematic random sampling based on probability proportional to size (PPS) method. List of HHs were obtained from the respective village’s administrative office. A total of 147 HHs were selected from the three villages using the formula proposed by Yemane (1967).

Both primary and secondary sources of data were used. Primary data were collected through HH survey, key informants’ interview and focus group discussions. Whereas, relevant secondary data were collected from different sources such as published and unpublished books and district’s agriculture and development office.

### Data analysis

Prior to conducting the data analysis, the HH questionnaires were coded and organised. The coding system was set up during the questionnaire preparation. Following the coding system, all the substantial HH questions were input in a coherent sheet of a Statistical Package for Social Sciences (SPSS) version 20 database to analyse the collected data. We used descriptive statics to analyse quantitative data on socioeconomic characteristics of HHs. Whilst the qualitative data gathered through FGD, KII and observational notes were transcribed, arranged and interpreted. Also, Chi-square tests were used in order to compare the difference amongst groups for different dependent variables. Furthermore, the multinomial logit (MNL) model was employed to identify the determinant variables that influence HHs’ adaptation strategies to climate change and variability, which is detailed in the following section.

### Econometric data analysis

In the present study, we used MNL model to identify the determinants of farmers’ adaptation decisions to climate variability and change. This model permits the analysis of decisions across more than two categories, allowing the determination of choice probabilities for different categories. However, the model requires that HHs are associated with only their most preferred option from a given set of adaptation strategies. The probability of using an adaptation measures by a given HH is independent of the probability of choosing another adaptation strategy. Thus, the model is specified as follows (Equations 1 and 2):

*Y* denotes a random variable with values (1, 2…J) for a positive integer *J* and *X* set of variables. In this study, *Y* is a dependent variable and represents the adaptation strategies (alternatives) from the set of adaptation measures, whereas *X* represents the factors that influence choice of the adaptation strategies, which contains HH attributes and *p*1, *p*2…*pj* as associated probabilities, such that *p*1 + *p*2 + … + *pj* = 1. This conveys how a certain change in *X* affects the response probabilities *p*(*y* = *j*/*x*), *j* = 1, 2 …J. As the probabilities must sum to unity, *p*(*y* = *j*/*x*) is determined once the probabilities for *j* = 2…J are known.

p(y=1x)=1−(p2+p3+…pj) [Eqn 1]

Furthermore, for a dependent variable with *j* categories, this requires the calculation of *j* − 1 equations, one for each category relative to the reference category, to describe the relationship between the dependent variable and the independent variables. The generalised form of probabilities for an outcome variable with *j* categories is:

$$p_{r}(y_{i}=j|\text{x})={\text{pr}}_{ij}=\frac{\exp(x\prime \beta \text{j})}{1+\sum_{j=1}^{j}\exp(x\prime \beta \text{j})},j=1,2,\ldots j$$ [Eqn 2]

The parameter estimates of the MNL model only provide the direction of the effect of the independent variables on the dependent (response) variable; estimates represent neither the actual magnitude of change nor the probabilities. Differentiating Equation 2 with respect to the explanatory variable provides the marginal effect of the independent variables which is given as:

$$\frac{\partial pi}{\partial xk}=pj\left(\beta jk-\sum_{j=1}^{j=1}pi\beta jk\right)$$ [Eqn 3]

The marginal effects or marginal probabilities are functions of the probability itself and measure the expected change in probability of a particular choice being made with respect to a unit change in an independent variable from its mean (Green 2000).

### Multicollinearity and autocorrelation test

In this study, we employed the variance inflation factor (VIF) technique to test multicollinearity amongst independent variables. Multicollinearity amongst explanatory variables can be reported if a VIF of 5 or 10 are detected (O’Brien 2007). Moreover, we used the Durbin−Watson test (*d*) to test autocorrelation. As *d* is approximately equal to 2 (1 − *r*), where *r* is the sample autocorrelation of the residuals, *d* = 2 indicated no autocorrelation (Durbin & Watson 1971).

### Hypothesised dependent and independent variables

The dependent variables, adaptation measures employed by farmers in the study area included crop diversification with improved varieties, income diversification, terracing for soil and water conservation, changing planting date, livestock diversification and fertilised application. The independent variables and their hypothesised effects are presented in Table 1. In this study, the adaptation theory was based on reviewing previous studies (Deressa et al. 2008, 2011; Legesse et al. 2013; Negash 2011; Tessema et al. 2013) and to validate the representativeness of these variables, we carried out focus group discussions with key informants.

**TABLE 1:** Description of independent variables and hypothesis for its effect on dependent variables.

Explanatory variables	Description	Expected sign
Gender	Dummy, 1 = male and 0 = female	±
Age	Continuous	±
Level of education	Continuous	+
Family size (active labour)	Continuous	+
Farm size	Continuous	+
Livestock ownership	Continuous	+
Total annual income	Continuous	+
Distance to the market	Continuous	−
Access to extension	Dummy, 1 = yes, 0 = no	+
Access to climate information	Dummy, 1 = yes, 0 = no	+
AEZs	Categorical, 2 = highlands, 1 = midland 0 = lowland	±

AEZ, agroecological zone.

### Ethical consideration

This article followed all ethical standards for research without direct contact with human or animal subjects.

## Results and discussions

### Socioeconomic and institutional characteristics of respondents

The study indicated that 86.4% HHs were male-headed HHs (25.2% from *lowland,* 23.1% from *midland* and 38.1% from *highland*) and the remaining 13.6% (3.4% from *lowland,* 4.8% from *midland* and 5.4% from *highland*) were female-headed HHs. In this study, a female-headed HH refers to a HH in which an adult woman (mostly with children) without male partner is the sole decision maker, whilst male-headed HH refers to a HH in which an adult man (mostly with a female partner and children) is the sole decision maker. The result is almost similar to the national coverage as reported by CSA (2012), which indicated that around 16% of the HHs in rural areas of the country are headed by women.

Furthermore, details regarding the age and family size of respondents showed that the youngest HH head was aged 27 years whilst the oldest was aged 81 years with a mean age of 49.14 years. Aged farmers can perceive the local climate condition and have higher probability to adapt the changing climate than younger farmers. The family size of HHs ranges from 1 to 12 members, with mean of five persons per HH and a s.d. of 1.50, which is similar with the reports of CSA (2012) that revealed that on an average, a HH in rural area of the country had about five individuals.

The climate resilience of smallholder farmers depends on access to natural resources such as farmlands and public services, including access to education and affordable credits (DFID 2011). The study indicated that the educational status of smallholder farmers ranges from 0 to 12 grade with mean of 1.89 and s.d. of 2.62. Of the total HH heads, about 60.5% of the respondents attended formal education and 39.5% of respondents did not attend formal education. The levels of literacy across the agroecological zonation of the district revealed that about 17.21% of HH heads in the lowland, 20.76% of HHs in the midland and 60.02% in the highlands were illiterates. Education level of HHs has substantial impact on the adoption of adaptation strategies to climate change. In this regard, Belay et al. (2017) indicated that educational levels of HHs need to be enhanced as it plays an important contribution to adopting adaptation measures and enhance agricultural production.

The results revealed that per capita farm size of farmers was small that falls between 0.125 ha and 1.25 ha and the average landholding was 0.4 ha per HH. In addition, 95.9% of the respondent possess less than 1 ha land size, whereas 4.1% had a land size greater than 1 ha. Livestock holding is one of the indicators of wealth status and an important component of farming system in the study area. The livestock holding of each HH was calculated in terms of tropical livestock unit (TLU) following Stocker et al. (1991). The HHs’ livestock ownership ranged from a minimum of 0.08 to a maximum of 7.21 TLU. On an average, the livestock holding of the sampled HH was 2.62 TLU. The annual income distribution of HHs ranged from 4000 to 22 600 Ethiopian Birr (ETB) with an average income of 11 232.41 ETB per year and a s.d. of 3578.75.

The study further indicated that about 93.2% of HHs have access to extension advice with frequency of extension contact ranging from 1 to 10 times per year. This showed that most of the HHs have better information, appropriate advice and technical support from development agents (DAs) on agricultural activities which could enhance their ability to adapt to climate-related shocks. This is supported by the reports by Birtukan and Abraham (2016) and Belay et al. (2017) who stated that HHs’ access to extension contact is likely to enhance their adoption of climate change adaptation strategies.

Moreover, majority of the HHs (97.3%) had access to credit service. This relaxes the financial constraints of farmers to adopt technology, which can enhance their climate resilience. However, most of the farmers (90%) had no access to climate information given from the stations. This suggests that the greater part of the rural farmers in the study area do not utilise the climate information given by the stations, which negatively influences them to do adaptation practices to climate change. Deressa et al. (2008) stated that farmers’ access to climate information can enhance and diversify the practices of adaptation strategies to climate change.

The results further revealed that 28.6% had access to market service, whereas 71.4% had no access. The average market distance of the respondents travelled to reach the nearest market centre was about 9.37 km with the minimum and maximum distance of 1 km and 18 km, respectively.

### Farmer’s adaptation strategies

During HH survey, respondents were asked whether there has been a practice of climate change adaptation strategies employed by farmers in the study area. Consequently, about 93.9 % of the HHs reported that they have employed various adaptation strategies to adverse effects of climate change and variability. However, the remaining 6.1% of the respondents have not done adaptation measures because of different reasons (see various predictors of adaptation strategies section). Similar results were reported by Belay et al. (2017) in their study in central rift valley region of Ethiopia who indicated that about 88% of the respondents were implementing various types of adaptation strategies to adverse impacts of climate change. The present result is also in agreement with the reports of Tessema et al. (2013) and Legesse et al. (2013) who indicated that the majority of the rural farmers in their study areas have employed different types of adaptation strategies to adverse impacts of climate change. This is obviously in line with the outcome acquired through focus group discussions and key informant interviews. However, the majority of the HHs complained that the ongoing adaptation practices are not adequate to reduce the adverse impact of climate change on their livelihood. This is on the ground that the majority of adaptation measures being carried out by rural farmers are generally represented by simple adaptation strategies.

**FIGURE 2:**
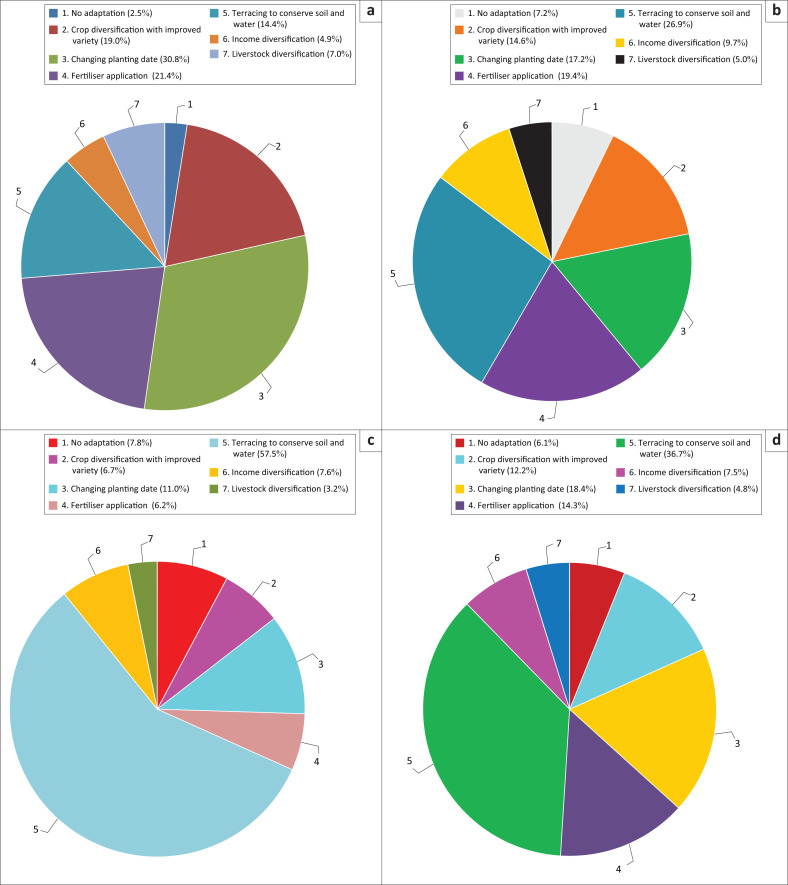
Adaptation strategies used by farmers in each agroecological zones and in the study area. (a) Lowland, (b) Midland, (c) Highlands and (d) in the district.

Besides, these HHs that implement climate adaptation activities were again asked to mention the major adaptation measures they employ against adverse impacts of climate change and variability. Accordingly, the adaptation strategies employed by rural HHs were terracing as soil and water conservation strategy (36.7%), changing planting date (18.4%), fertiliser application (14.3%), crop diversification (12.2%), income diversification (7.5%) and livestock diversification (4.8%; Figure 2). The fact that majority of the HHs practice terracing as soil and water conservation strategy is an indication that the areas are highly vulnerable to soil erosion. This indicates that terracing was the most common adaptation strategy used by the farmers in study area as compared with other adaptation strategies to tackle the adverse effect of climate variability in crop production.

The adaptation strategies of rural farmers were compared by agroecological zone (lowland, midland and highland communities). The results indicated that the types of adaptation strategies employed by farmers were similar in both study sites but the difference was priority of the strategies they used. For instance, the first priority considered by farmers in lowland area was changing planting date (30.8%) followed by fertiliser application (21.4%) and crop diversification (19%) whereas in highland area terracing (57.5%) was the first priority of farmers to reduce the effect of soil erosion and runoff on crop production followed by changing planting date (11%), crop diversification (7.8%) and income diversification (7.6%). Similar to highland farmers, terracing was also midland farmer’s priority (36.7%) followed by changing planting date (18.4%), fertiliser application (14.3%) and crop diversification (12.2%) to adapt adverse impacts of climate-related risks (Figure 2). Similar results were reported by Hirpha (2020) who indicated that diversifying crops, terracing and early planting and income diversification were the main adaptation practices employed by rural farmers in Adama district, central rift valley region of Ethiopia. However, some of the strategies reported by the same author such as changing quantity of land under cultivation and irrigation crop farming were different from the adaptation strategies implemented in our study sites. This shows that some climate change adaptation measures are location-specific. This is supported by the finding of Hinkel (2011) who indicated that climate change adaptation measures were area-specific and affected the socioeconomic condition.

### Barriers to adaptation strategies

**FIGURE 3:**
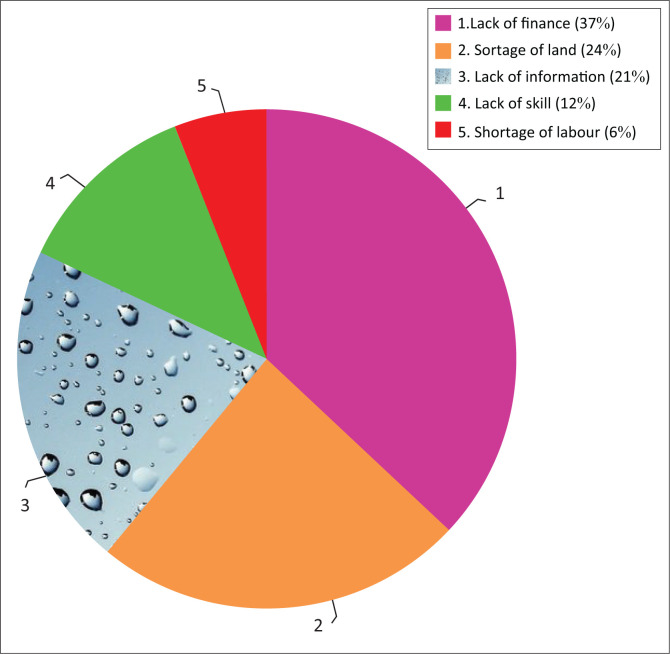
Barriers to climate change adaptation in the study area.

The respondents were asked to answer the barriers to adaptation. The most important factor mentioned as barrier to adaptation by the surveyed farmers were lack of finance (money), lack of climate information, shortage of land, lack of skill and shortage of labour (Figure 3). The majority of respondents (37%) reported that lack of money was one of the main barriers to hinder farmer’s adoption of climate change adaptation measures. Money is essential to purchase agricultural inputs such as irrigation equipment, tools for soil and water conservation, improved crop and livestock variety. Lack of money hinders farmers from getting the necessary resources and technologies that facilitate adapting to climate variability and change. About 24% of HHs perceived shortage of farmland as a barrier to climate change adaptation. Land is an important agricultural asset that helps farmers to reduce climate-related risks through crop diversification and use of improved crop varieties. This is in line with the reports of Bryan et al. (2013) and Abid et al. (2015) who revealed that rural farmers with large land size have more capacity to adapt adverse effects of climate change through the use of improved crop varieties. Moreover, 21% of the local respondents complained that lack of access to climate information was one of the barriers that hinders farmers’ adoption of climate change adaptation measures. The results revealed lack of knowledge and skills about suitable adaptation measures amongst barriers that limit farmers’ adoption of climate risk reduction measures as reported by 12% of HHs. This result is supported by the findings of Abid et al. (2015) and Kide (2014) who showed that lack of climate information, lack of knowledge and money were the major limitations to hinder rural HHs’ willingness to adopt climate change adaptation measures. Moreover, shortage of labour, as reported by 6% of farmers, was the least barriers to climate change adaptation. Similar results were reported by Tessema et al. (2013), Belay et al. (2017), Mekonnen (2018) and Nega et al. (2019) who indicated lack of money, lack of information, inadequate labour and shortage of land as major barriers to adaptation measures.

### Determinants of farmer’s choice of adaptation strategies

Estimated parameter estimates of multinomial logit climate change adaptation model are indicated in Table 2. The explanatory variables are categorised as demographic, human capital, assets and income, access to public services and agroecological zone (Table 3). The results indicated that all explanatory variables except gender significantly affect the adaptation strategies. In the following section, only the variables that were statistically significant at less than or equal to 10% probability levels are interpreted and discussed.

**TABLE 2:** Estimated parameter estimates of multinomial logit climate change adaptation model.

Explanatory variable	Terracing for soil & water conservation			Fertiliser application			Income diversification			Livestock diversification with supplementary feed			Changing planting date			Crop diversification with improved variety		
	Coeff.	s.e.	*p*-value	Coeff.	s.e.	*p*-value	Coeff.	s.e.	*p*-value	Coeff.	s.e.	*p*-value	Coeff.	s.e.	*p*-value	Coeff.	s.e.	*p*-value
Gender	1.1515	1.3990	0.410	2.6372*	1.5581	0.091	−1.1839	2.7575	0.668	3.1461*	1.8132	0.083	0.8470	1.7212	0.623	3.5734	2.1584	0.098
Age	0.1640	0.1009	0.104	0.1819*	0.1071	0.090	−0.7324	0.5001	0.143	0.2151*	0.1215	0.077	0.3464***	0.1114	0.002	0.0716	0.1536	0.641
Education	0.6886	0.6058	0.256	−0.8628	0.6917	0.212	−2.1016	2.0427	0.304	1.5305*	0.8417	0.069	0.0762	0.7384	0.918	6.9736***	2.3363	0.003
Family size (active labour)	−2.0905**	0.9434	0.027	−1.8004*	0.9708	0.064	−2.7241*	1.4273	0.056	−1.9739*	1.0484	0.060	−1.4513	0.9639	0.132	1.9666	1.4074	0.162
Farm size	−0.0683	3.4735	0.984	2.2337	3.6994	0.546	−20.3211*	11.7864	0.085	2.5461	4.6052	0.580	−1.7347	3.5822	0.774	1.5942	5.6091	0.776
TLU	2.771***	0.9777	0.005	−1.5232	0.9566	0.111	4.2992***	1.3545	0.002	−0.0738	1.1384	0.948	−1.3803	0.9619	0.151	−1.3841	1.0973	0.207
Income	0.0006*	0.0003	0.072	0.0014***	0.0004	0.001	0.0023	0.0016	0.157	0.0011***	0.0004	0.008	0.0013***	0.0004	0.001	0.0021***	0.0005	0.000
Market	−0.3736	1.3604	1.3604	−0.0005*	1.5406	0.100	−1.944068	2.8284	0.492	−0.9918	1.7388	0.568	−0.1366	1.5018	0.928	−1.3630	2.1018	0.517
Information	0.5495	1.8944	0.772	1.4172	1.9596	0.470	5.9488	4.1052	0.147	1.4358	2.2168	0.517	2.1852	1.9886	0.272	0.1518	2.6670	0.955
Extension	1.0414	1.4192	0.463	−0.2460	1.9316	0.899	−0.4246	4.1755	0.919	1.6083	2.6684	0.547	1.0692	1.7350	0.538	6.0854	4.3918	0.166
AEZ (Mid)	2.5177	3.3764	0.456	−1.7139	3.3708	0.611	−7.02196	5.1316	0.171	−1.6637	3.5686	0.641	−1.8589	3.4111	0.586	−6.1070	4.0987	0.136
AEZ (High)	1.5711	3.3429	0.638	−6.3630*	3.5956	0.086	−6.5242	4.8296	0.177	−5.5542	3.7823	0.142	−8.1922**	3.7101	0.026	4.9722	4.1267	0.228

Coeff., coefficient; s.e., standard error.

Base category = No adaptation; Number of observations = 147; LR *χ*^2^ (78) = 312.51; Log likelihood = -97.2101; Prob > *χ*^2^ = 0.0000; Pseudo *R*^2^ = 0.6165.

***, **, * , significant at 1%, 5% and 10% probability level, respectively.

**TABLE 3:** Marginal effects from the multinomial logit climate change adaptation model.

Explanatory variable	Terracing for soil & water conservation		Fertiliser application		Income diversification		Livestock diversification		Changing planting date		Crop diversification with improved variety		No adaptation	
	dy/dx	s.e.	dy/dx	s.e.	dy/dx	s.e.	dy/dx	s.e.	dy/dx	s.e.	dy/dx	s.e.	dy/dx	s.e.
**Demographic**														
Gender	−0.0159	0.0626	0.0928	0.0688	−0.0310	0.0223	0.0466	0.0470	−0.0861	0.0598	0.0356	0.0386	−0.0419	0.0348
Age	0.0145*	0.0076	−0.0007	0.0030	−0.0163**	0.0076	0.0014	0.0021	0.0130***	0.0026	0.0067**	0.0030	−0.0051	0.0033
Family size	−0.0592	0.0415	−0.0026	0.0338	−0.0157	0.0201	−0.0111	0.0183	0.0220	0.0275	−0.0046	0.0308	−0.0714**	0.0320
**Human capital**														
Education	0.0148	0.0381	−0.0581*	0.0212	−0.0507	0.0312	0.0031	0.0184	0.0575**	0.0272	0.1671***	0.0435	−0.0287	0.0184
**Assets and income**														
Farm size	0.2574	0.2178	0.2061	0.1699	0.2343	0.1631	0.0647	0.1100	−0.1947	0.1327	0.3103**	0.1241	0.0304	0.1249
Livestock holding	0.12547***	0.0302	0.0083	0.01975	0.0346***	0.0114	0.0485**	0.0238	0.0168	0.0191	0.0041	0.0182	0.0821	0.0404
Income	8.8444	0.00002	0.00002***	8.8222	0.00005**	0.00002	−4.5444	0.5888	0.00002***	7.7555	0.00002***	9.0111	−0.00001**	0000
**Public access**														
Access to market	0.0110	0.0738	0.0441	0.0611	−0.0298	0.0449	−0.0234	0.0347	0.0103	0.0467	−0.01291**	0.0440	0.0168	0.0472
Climate information	0.1102*	0.0629	0.0181	0.0628	0.0780***	0.0252	0.0079	0.0472	0.0804	0.0552	−0.0319	0.0479	−0.0423	0.0497
Extension access	0.1494*	0.0887	−0.0780	0.0714	0.0064	0.0753	0.029819	0.0855	0.0672	0.0797	0.1192***	0.0462	0.0047	0.0444
**Agroecological zonation**														
Midland	0.1185***	0.0802	0.0760	0.0909	−0.0845**	0.0375	0.0229	0.0562	0.0142	0.0927	−0.0902***	0.0327	0.0430	0.0330
Highland	0.3793***	0.0777	−0.1146*	0.0588	−0.0874***	0.0338	−0.0108	0.0407	−0.2003***	0.0580	−0.0256	0.0560	0.0596*	0.0317

dy/dx, marginal effect; s.e., standard error.

***, **, * , indicates significant at 1%, 5% and 10% probability level, respectively.

#### Demographic

The results indicated that the age of the HH heads had significant positive and negative effects on the choices of adaptation strategies. Specifically, the results show that age of the HH was found to be positively and significantly correlated with terracing, changing planting date and crop diversification with improved variety at *p* ≤ 0.1, 0.01 and 0.05, respectively, compared with the base category. This means that an increase in the age of HH head by 1 year increases the probability of the farmers practicing terracing as soil and water conservation strategy by 1.4%, changing planting date by 1.3% and crop diversification with improved variety by 0.06%. This could be explained by aged farmers who are assumed to have better knowledge about the local climate and farming experience. Therefore, they can easily adjust themselves to climate-related shocks. The findings are similar with the reports of Marie et al. (2020) who indicated that a unit increase of a HH head significantly increases the probability of HH head using improved crop varieties and mixed cropping by 1.3% and 1.9%, respectively, in their study in north-western Ethiopia.

The result is consistent with Quayum and Amin (2012), Birtukan and Abraham (2016) who reported that older farmers are more likely to involve crop-diversification than younger ones as adaptation strategy. However, the present finding is in contradiction with Haftu et al. (2016) who reported a negative correlation of being aged with soil and water conservation practices. On the other hand, age of the HH head showed a unit increase in the age of the HH decreases the probability of farmers using income diversification such as off-farm income sources by 1.6% at *p* ≤ 0.5 as an adaptation strategy.

Moreover, as expected, the MNL model revealed that family size is positively and significantly correlated with farmers’ adoption of terracing practice, fertiliser application, income diversification, livestock diversification, changing planting date and crop diversification. A one-unit increase in the family size increases the probability of farmers to use these adaptation strategies by 7.1% at *p* ≤ 0.05. This indicates those HHs who have large active labours who have an opportunity of pursuing various adaptation options in the face of adverse impacts of climate change. This is consistent with Menberu and Yohannes (2014) who argued that large family is associated with higher labour endowment, which would enable a HH to accomplish various adaptation strategies. The present findings are in line with the findings of Marie et al. (2020) who showed that a unit increase in the family member of a farmer resulted in 2%, 3.1% and 1.6% increase in the probability of farmers using improved crop varieties, soil conservation technique and mixed cropping, respectively, as adaptation strategy. This finding is also agreeable with finding of Gebrehiwot and Veen (2013) who revealed that farmers with large family size have higher probability of implementing adaptation measures than their counterparts. A positive and significant association between family size of a farmer and climate change adaptation strategies has also been found in several studies (Abid et al. 2015; Ali & Erenstein 2017).

#### Human capital

Level of education significantly affected the choices of adaptation strategies to climate variability and change. A unit increase in education increases the likelihood of adopting crop diversification with improved variety by 16% (*p* ≤ 0.01) and change in planting date by 5.7% at *p* ≤ 0.05. This might be because of the fact that literate farmers are more aware to adopt new technologies compared with their counterparts. The result is agreeable with the findings of Hirpha (2020) who reported that literate HH heads are more responsive concerning their farming technologies and they have better access to scientific information compared with the illiterate farmers. This result is supported by Ali and Erenstein (2017) who reported that educated farmers are likely to be more aware of climate change and agricultural innovations and may be more interested in adopting technology and methods to adapt adverse climate change impacts.

#### Farmers’ assets and income

Recent study has indicated that farmers’ assets affect the implementation of farm technology (Mmbando and Baiyegunhi 2016). In the present study, positive association was observed between farm size and adoption of farmers’ crop diversification with improved variety. A unit increase in farm size increases the probability of using crop diversification with improved variety by 31% (*p* ≤ 0.05). The findings are supported by the findings of Ojo and Baiyegunhi (2018) who showed statistically significant association between farm size and implementation of planting improved varieties.

Moreover, the results indicated that the size of livestock holding is found to affect positively and significantly the use of terracing as soil and water conservation strategy, income diversification and livestock diversification. A unit increase in number of livestock increases the probability of adopting terracing as soil and water conservation strategy by 12% at *p* ≤ 0.01, income diversification by 3.4% at *p* ≤ 0.01 and livestock diversification by 4.8% at *p* ≤ 0.05. This could be explained by those farmers with large herd size who have better chance to earn more money to invest on tools required for conservation practices, alternative income sources and for livestock feeds. Similar results were reported by Belay et al. (2017), Deressa et al. (2011), Haftu et al. (2016) and by Ali and Erenstein (2017) who revealed statistically significant relationship between livestock ownership and adaption of sowing time adjustment and crop diversification.

The results further showed that farmers’ adaptation strategy to climate change is also significantly affected by the HHs’ income level. A unit increase in the total income of HHs increases the probability of adopting fertiliser application by 0.002%, income diversification by 0.005%, changing planting dates by 0.002% and crop diversification with improved variety by 0.002% at *p* ≤ 0.01. The reason might be that farmers with higher income may have additional financial power to invest on different adaptation strategies. The results are supported by Abid et al. (2015), Iheke and Agodike (2016) and Saguye (2016) who revealed positive and significant association between income level and the implementation of climate change adaptation strategies. This shows that HHs with better income level are more likely to employ climate change adaptation measures than their counterparts. Similar results were reported by Ali and Erenstein (2017) who indicated that wealthy farmers have better chance to adopt new agricultural technology to cope with the adverse impacts of climate change. The authors reported positive and significant relationship between income level and adoption of sowing time adjustment, drought tolerant varieties and crop diversification.

#### Access to public services

The results indicated that as expected, distance to market has negative and significant relation with crop diversification and improved variety at *p* ≤ 0.05 level of probability. A HH that resides near to market has higher likelihood of using different crops with improved varieties by 1.2% than those farmers far from market access. The findings are supported by Marie et al. (2020) who reported that HHs with market access have higher probability to adopt climate change adaptation measures than HHs with lack of market access. Similar results were reported by Hassan and Nhemachena (2008) who indicated that market access creates an opportunity to farmers to grow and thereby improving the income and climate resilience of farmers. Moreover, Bryan et al. (2009) and Belay et al. (2017) reported that HHs with market access have a great chance to look for alternative climate change adaptation strategies.

Furthermore, access to climate information had positive influence on the probability of adopting adaptation options to climate variability and change. Farmers’ access to climate information increases the probability of using terracing as soil and water conservation strategy by 11% at *p* ≤ 0.1 and income diversification by 7.8% at *p* ≤ 0.01. This is because of the fact that climate information access enhances HHs’ awareness and knowledge of the changing local climate and the climate change adaptation measures. Similar results were reported by Hirpha et al. (2020) and Tazeze et al. (2012) who revealed that access to climate information was found to be positive and it significantly influenced the implementation of adaptation measures. This result was in line with the findings of Balew et al. (2014) and Negash (2011) who revealed that access to climate information had significant and positive relation with adoption of farmers’ climate change adaption strategies.

Extension services are critical to enhance farmer’s knowledge and skills that increase adoption of improved agricultural technology including climate resilience practices. According to Bryan et al. (2013), farmers who did not have access to extension services are more likely to either not perceive climate change or perceive it wrongly. Results showed that, a unit increase in extension contact is likely to increase the probability of the farmer to practice terracing as soil and water conservation strategy by 14% at *p* ≤ 0.1 and crop diversification with improved crop varieties by 11% at *p* ≤ 0.01. This is because farmers with more extensive access and technical assistance on agricultural activities create more awareness to adopt adaptation strategies. The results are in agreement with the findings of Ojo and Baiyegunhi (2018) who revealed that extension access is positively related with farmers’ adoption of improved crop varieties, crop diversification and sowing time adjustment. Similarly, Abid et al. (2015) indicated positive and statistically significant association between farmers’ access to extension and implementation of mixed cropping and using improved varieties as climate change adaptation strategies.

#### Agro-ecological zone

Climate change has location-specific impacts on agricultural production and hence, farmers will have location-specific adaptation response to adverse impacts of climate change (Below et al. 2012; Hinkel 2011). The present study tested if there is significant association between agroecological zones (lowland, midland and highland areas) and climate adaptation measures employed by the local farmers in the study sites. The results showed that the probability of using terracing for soil and water conservation and crop diversification with improved varieties increased by 11% at *p* ≤ 0.01 and 9% at *p* ≤ 0.01, respectively, with midland agroecological zone compared with lowland area. Moreover, farmers located in the highland agroecological zone are 37% more likely to use terracing as soil and water conservation strategy. This means that farmers in the *highlands* and *mid-highlands* are more likely to choose cultivation of different crops and terracing as soil and water conservation measures compared with those in the *lowlands*. This might be explained by farmers who experienced various types and intensity of climate-induced shocks because of their variation in agroecological setting and hence responded differently. The results are supported by Komba and Muchapondwa (2012) who indicated that the likelihood of using crops that are drought resistant decreases with location of the plot in agroecological zones other than arid. However, the probability of using short-season crops relative to no adaptation increases with location in the costal agroecological zone.

Moreover, the present result is in agreement with Birtukan and Teichman (2010), Wondimagegn and Lemma (2016) who reported that different farmers living in different agroecological settings employ different adaptation methods to the effects of climate variability and change. The result further indicated that farmers in the *highland* zone significantly decrease the probability of using fertiliser application by 11% and income diversification by 8.7% (*p* ≤ 0.01) as compared with farming in *lowlands*. Moreover, farmers in *highlands* significantly reduce the probability of changing planting date by 20% (*p* ≤ 0.1).

## Conclusion and recommendations

The results indicated that majority of the smallholder farmers employed different adaptation strategies to adapt to adverse effects of climate change and variability, including terracing as soil and water conservation strategy, changing planting date, fertiliser application, crop diversification with improved variety, income diversification and livestock diversification. The results further revealed that although the local people employed different strategies to adapt the adverse effects of climate-induced shocks, there were constraints that limit the HH’s adaptation strategies. These include lack of finance, limited technical skills and lack of climate information. The result further showed that age, family size, farm size, educational level, income, livestock holding, access to extension services, distance to market and agroecological zones determined farmers’ choice of adaptation strategies.

Therefore, the local decision makers such as agricultural sector, microfinance sector, meteorological agency should provide farmers with credit access and climate information access to reduce shortage of finance and lack of climate information. There is also a need to provide farmers with training on improved agricultural technology and market access to enhance their climate resilience. There is a need to promote appropriate adaptation measures for a particular agroecological zone. For example, in midland and highland agroecological zones, farmers are more likely to use crop diversification with improved varieties and terracing as soil and water conservation strategy. Hence, it is critical to promote these adaptation strategies to reduce the adverse impacts of climate variability in this study’s sites.
